# Proteomics-Based Identification of Differentially Abundant Proteins from Human Keratinocytes Exposed to Arsenic Trioxide

**DOI:** 10.4172/jpb.1000317

**Published:** 2014-07

**Authors:** Udensi K Udensi, Alan J Tackett, Stephanie Byrum, Nathan L Avaritt, Deepanwita Sengupta, Linley W Moreland, Paul B Tchounwou, Raphael D Isokpehi

**Affiliations:** 1 RCMI Center for Environmental Health, College of Science, Engineering and Technology, Jackson State University, Jackson Mississippi 39217, USA; 2 Proteomics Facility, University of Arkansas for Medical Sciences, Department of Biochemistry and Molecular Biology, Little Rock, AR 72205, USA; 3 Department of Biology, School of Science, Engineering and Mathematics, Bethune-Cookman University, Daytona Beach FL 32114, USA

**Keywords:** Arsenic, HaCaT keratinocytes, Skin cancer, TFRC, Proteomics, Visual analytics

## Abstract

**Introduction:**

Arsenic is a widely distributed environmental toxicant that can cause multi-tissue pathologies. Proteomic assays allow for the identification of biological processes modulated by arsenic in diverse tissue types.

**Method:**

The altered abundance of proteins from HaCaT human keratinocyte cell line exposed to arsenic was quantified using a label-free LC-MS/MS mass spectrometry workflow. Selected proteomics results were validated using western blot and RT-PCR. A functional annotation analytics strategy that included visual analytical integration of heterogeneous data sets was developed to elucidate functional categories. The annotations integrated were mainly tissue localization, biological process and gene family.

**Result:**

The abundance of 173 proteins was altered in keratinocytes exposed to arsenic; in which 96 proteins had increased abundance while 77 proteins had decreased abundance. These proteins were also classified into 69 Gene Ontology biological process terms. The increased abundance of transferrin receptor protein (TFRC) was validated and also annotated to participate in response to hypoxia. A total of 33 proteins (11 increased abundance and 22 decreased abundance) were associated with 18 metabolic process terms. The Glutamate--cysteine ligase catalytic subunit (GCLC), the only protein annotated with the term sulfur amino acid metabolism process, had increased abundance while succinate dehydrogenase [ubiquinone] iron-sulfur subunit, mitochondrial precursor (SDHB), a tumor suppressor, had decreased abundance.

**Conclusion:**

A list of 173 differentially abundant proteins in response to arsenic trioxide was grouped using three major functional annotations covering tissue localization, biological process and protein families. A possible explanation for hyperpigmentation pathologies observed in arsenic toxicity is that arsenic exposure leads to increased iron uptake in the normally hypoxic human skin. The proteins mapped to metabolic process terms and differentially abundant are candidates for evaluating metabolic pathways perturbed by arsenicals.

## Background

Arsenic (As) is a widely distributed environmental toxicant that causes skin cancer [1]. The types of skin cancer associated with arsenic include intraepidermal carcinomas (Bowen disease) [2], squamous cell carcinomas (SCC), basal cell carcinomas (BCC) [3], Merkel cell carcinoma (MCC) [4] and head and neck cancers [5]. Microarrays gene expression has revealed that arsenicals impact the function of diverse tissue types including the skin, [6,7], bladder and kidney [8], liver [9], prostate and lung [10], peripheral lymphocytes [11], neural tube [12], and urogenital cells [13]. Arsenic also is useful for the treatment of relapsed or refractory acute promyelocytic leukemia as well as solid tumors [14–17]. Clearly, diverse tissue types are affected by arsenic. However, new knowledge is needed to understand the molecular mechanisms of arsenic-induced cancers and arsenic-treatment of cancers in diverse tissue types.

The objective of the research reported here was to determine proteins in HaCaT keratinocyte that are altered in response to arsenic exposure. The application of toxicogenomics methods including microarray gene expression has facilitated the understanding of molecular pathology, and increased the understanding of toxicant responses [18,19]. Toxicoproteomics uses high-throughput technologies to identify critical proteins and pathways in biological systems that are affected by adverse chemical and environmental exposures. Therefore, to reliably identify proteins perturbed by arsenic trioxide in HaCaT keratinocyte model, we have used a combination of LC-MS/MS with a Thermo LTQ-XL mass spectrometer coupled to an Eksigent nanoLC-2D [20].

The results from proteomics experiments provides list of proteins with altered abundance that have statistical significant changes in their abundance between two conditions. The list of proteins presents data set for discovery of functional categories that could be of biological significance to the arsenic-induced pathologies. For example, skin is a target organ for several pathological manifestations of arsenic toxicity including both hypo- and hyperpigmentation [21]. Several bioinformatics tools enable the annotation of the list of proteins with functional categories [22–36]. A challenge with multiple heterogeneous functional annotations is availability of informatics resources for conducting a variety of tasks on the data sets. These tasks include identifying groups of altered proteins with identical functional categories.

In our research, the data obtained from proteomics were annotated for tissue types [22,26–28]. Gene Ontology [28,32], and gene family membership [33]. A visual analytics environment was developed to facilitate several tasks designed to construct knowledge on potentially arsenic-modulated (i) tissue types; (ii) biological processes; and (iii) gene families. Visual analytics environments provide software and hardware to interact with data as well as make sense of relationships and patterns in collection of data sets [34].

In this report, arsenic-regulated proteins refer to proteins from the proteomics experiments with altered abundance (spectral counts) in the HaCaT keratinocyte exposed to arsenic relative to the control condition (no exposure to arsenic). The terms for describing proteins with increased abundance and proteins with decreased abundance were up-regulation and down-regulation respectively. The abundance of 173 proteins was altered in keratinocytes exposed to arsenic; in which 96 proteins had increased abundance while 77 proteins had decreased abundance. These proteins with altered abundance were also classified into 69 Gene Ontology biological process terms. Based on our prior research [35,36], we expected the up-regulation of the human transferrin receptor protein (TFRC) in the proteomics experiment. TFRC is involved in cellular uptake of iron via receptor-mediated endocytosis of ligand-occupied transferrin receptor into specialized endosomes [37–39]. We have identified a need for additional research on TRFC involvement in the hyperpigmentation observed in arsenic induced diseases [36]. Indeed, an increased abundance of transferrin receptor protein (TFRC) was observed and validated with RT-PCR and western blot. Further, TFRC was annotated to participate in response to hypoxia. A possible explanation for hyperpigmentation pathologies observed in arsenic toxicity is that arsenic exposure leads to an increased iron uptake and accumulation in the normally hypoxic human skin [37]. A total of 33 proteins (11 increased abundance and 22 decreased abundance) were associated with 18 metabolic process terms. The proteins mapped to metabolic process terms and differentially abundant are candidates for evaluating metabolic pathways perturbed by arsenicals.

## Method

### Cell culture

HaCaT cells (obtained from Dr. Van Wilson, Microbial & Molecular Pathogenesis, College of Medicine, Texas A&M Health Science Centre, College Station, Texas, USA) were cultured in Dulbecco’s Modified Eagle’s medium (DMEM) media supplemented with 10% fetal bovine serum (FBS) and 100 μg/mL penicillin-streptomycin at 37°C with 5% CO_2_ in a humidified incubator. The cell lines were divided into two experimental groups; Exposed and Unexposed. Each group had three biological replicates. The exposed cells were exposed to 0.5 ppm of arsenic trioxide and passaged up to 8 passages. The unexposed controls were also passaged (passage control) alongside the exposed groups but without any exposure to arsenic trioxide up to passage 8 [40].

### Cytotoxicity assay (MTT Assay)

Dose response relationship was determined by measuring the survival rate (% viability) of cells after treatment with arsenic trioxide using MTT (tetrazolium salts) assay. Arsenic trioxide (arsenic trioxide dissolved in dilute nitric acid, 1 mL=1 mg AS; Reference Standard Solution, 1000 ppm ± 1%/certified, 99.9% purity, Fisher Scientific) dilutions were prepared using a complete DMEM medium as the diluent. Using a 96 well plate, a total of 200 μL complete medium only was added to the first column, then 100 μL of HaCaT cell suspension in complete medium containing approximately 20,000 cells/well was added to columns 3–8 wells of the 96-well plates, 100 μL of medium was added to column 2, and 100 μL of arsenic trioxide concentrations was added to columns 3–8, ensuring that the final concentrations were 0, 1.0, 5.0, 10, 15, 20, and 25 ppm, respectively. They were incubated in a humidified incubator at 37°C in a 5% CO_2_ for a period of 48 hours. 10 μL of the yellow MTT reagent was added to each of the wells and incubated at 37°C for 4 hours until purple formazan precipitate was visible. Then 100 μL of the detergent reagent was added to each of the wells and kept in the dark at room temperature for 2 hours. The absorbance was measured at 570 nm wave length using a microtiter plate reader (Fluoroskan II microplate reader, Helsinki, Finland). LD_10_ and LD_50_ were determined from the dose-response curve. Acute cytotoxicity experimental results were used to determine tolerated dose (LD_10_) in all the cell lines for chronic assays as used by Trouba et al. [41].

### Cell harvest

At approximately 80% confluence in DMEM/10% FBS at 37°C with 5% CO_2_, the media was discarded and washed three times with 5 mL DPBS. About 2 mL of 0.25% trypsin EDTA (1x) (Gibco, Life Technologies) was added to dissociate the cells and 4 mL of whole media was added to stop the trysinization reaction. The supernatant with the dissociated cells were completely aspirated into 15 mL centrifuge tubes and centrifuged at 300 g for 5 minutes. The media was aspirated out and the pellet was washed 3 times with 5 mL DPBS.

### Cell lysis

The pellets were resuspended in 150 μL of 2X BME (β-mercaptoethanol) (155.8 mM tris-HCL, 0.006% bromophenol blue, 49.8 mM tri base, 12.5 mM TCEP-HCl, 25% glycerol, 5% SDS) in 1.5 mL microcentrifuge tube. It was sonicated for 5 minutes using a Bioruptor UCD 200 (Diagenode) on high power with a 30 second on/off cycle and heated at 95°C for 5 minutes and finally centrifuged at max for 1 minute.

### Determination of protein concentration

The protein concentration in the cell lysate was measured by spotting 2 μl of each sample unto read plate and was read with Take Three BioTek microplate spectrophotometer. It was analyzed using Take Three interface and the 280 nm reading was used to approximate loading volumes.

### Determination of proteomic responses

Protein was resolved using SDS-PAGE. Each SDS-PAGE gel lane was sliced into 24 bands of 3 mm segments in a laminar flow hood. Enzymatic digestion of proteins was performed by (i) reduction of disulfide bonds using Tris (2-carboxyethyl) phosphine hydrochloride (TCEP), (ii) akylation of reduced Cys residues with iodoacetamide (IA) and (iii) digestion of proteins with trypsin. The reaction was stopped by adding formic acid. Tryptic peptides from the 144 gel bands were analyzed by nanoflow LC-MS/MS with a Thermo Orbitrap Velos mass spectrometer equipped with a Waters nanoACQUITY LC system. Tryptic peptides were separated by reverse phase Jupiter Proteo resin (Phenomenex) on a 100×0.1 mm column using a nanoAcquity UPLC system (Waters). Peptides were eluted using a 30 min gradient from 98:2 to 40:60 buffer A:B ratio. [Buffer A=0.1% formic acid, 0.05% acetonitrile; buffer B=0.1% formic acid, 75% acetonitrile.]

Eluted peptides were ionized by electrospray (2.0 kV) followed by MS/MS analysis using collision induced dissociation on a Thermo LTQ Orbitrap Velos mass spectrometer. MS data were acquired using the FTMS analyzer in profile mode at a resolution of 60,000 over a range of 375 to 1500 m/z. MS/MS data were acquired for the top 15 peaks from each MS scan using the ion trap analyzer in centroid mode and normal mass range with normalized collision energy of 35.0. Proteins were identified by a Mascot (version 2.3.01) database search with the following parameters: precursor ion tolerance 5 ppm, fragment ion tolerance 0.65 Da, fixed modification of carbamidomethyl on cysteine, variable modification of oxidation on methionine and acetylation on n-terminus, and 2 missed cleavages possible with trypsin. We searched the human ‘Con_IPI_Hum.v3.34a’ database (International Protein Index for *Homo sapiens*) and we additionally used reversed sequences for higher confidence identifications. The Mascot results were uploaded into Scaffold 3 (version 3.5.1) for viewing the proteins and peptide information. A protein threshold of 95%, minimum of 2 peptides, and a peptide threshold of 50% were used to export spectral counts to spreadsheet file for further data analysis. The general rule is to explain the spectral data with the smallest set of proteins when using scaffold to determine peptides that could be assigned to different proteins, hence our application of a 50% peptide threshold. For example, if protein A and protein B each have one unique peptide, they will be listed separately only if the peptide probability is >50% [42].

### Identification of arsenic regulated proteins

A label-free approach based on spectral counting was used [43,44] to determine if a protein was differentially regulated between arsenic treated and the untreated control cells. Spectral count by definition is the number of tandem mass spectra assigned to a given protein in all bands from a single gel lane. To determine the relative amount of a protein in a given gel lane, a normalized spectral abundance factor (NSAF) was calculated [44]. NSAF is calculated as the number of spectral counts (SpC) identifying a protein, divided by the protein’s length (L), divided by the sum of SpC/L for all proteins in the experiment. The abundance of individual proteins in multiple independent samples can be compared using NSAF, which also allows quantification of expression changes in different complexes [45,46].

Prior to statistical analysis, the spectral count data were first normalized in order to compare between samples, account for heteroscedasticity (log transformation), and to account for the relative amount of proteins in a given gel lane by calculating a Normalized Spectral Abundance Factor (NSAF) [44,47].

$${\left(\mathrm{NASF}\right)}_{k}=\frac{(\frac{\mathrm{SpC}}{MW})k}{\sum_{i=1}^{N}{\left(\frac{\mathrm{SpC}}{MW}\right)}_{i}}$$

The NSAF for a protein *k* is the number of Spectral Counts (SpC) identifying a protein, *k*, divided by the protein’s Molecular Weight (MW), divided by the sum of SpC/MW for all N proteins in the gel lane [20].

Different proteins were identified among the arsenic exposed and unexposed keratinocyte cells. Therefore, some proteins will contain a spectral count of zero for a particular sample. In order to allow for log transformation, Zybailov et al. [44] described a method for processing spectral counts of zero in the data set by replacing the zero values with a fractional value prior to the NSAF calculation. In the present study, we replaced zero spectral count values with 0.5. As is commonly done in microarray data sets, the data was scaled to ensure the medians of all distributions were equal and centered to ensure the standard deviations of all distributions were equal, allowing for more robust statistical testing [29]. The significance of the differences in spectral abundance was determined with t-tests.

### Real Time RT-PCR and western blots of transferrin receptor (TFRC)

In our prior bioinformatics research, we observed that arsenic increased the expression of the genes for transferrin receptor [35,36]. To confirm this prediction, we conducted RT-PCR and Western Blot experiments. Total RNA from cultured cells was extracted using RNeasy Mini kit (Qiagen, Valencia, CA) following the manufacturer’s specifications. RNA was treated with RNase-free DNase I Amplification Grade (Invitrogen, Grand Island, NY) to remove any DNA contamination. About 1 μg of purified RNA was used for synthesis of cDNA with oligo dT primers using Superscript First-Strand Synthesis System (Invitrogen, Grand Island, NY) following manufacturer’s instructions. The cDNA was purified by RNase H (Invitrogen, Grand Island, NY). Quantitative real-time PCR was performed using a reaction mixture containing 1X Advanced SYBR Green Supermix (Bio-Rad, Hercules, CA), 0.33 μM of each primer. The cyclic conditions used were 95°C for 2 min, followed by 45 cycles at 95°C for 15 s and 50°C for 20 s. The following primer pairs were used: TFRC; Forward Primer: 5′-GCGATAACAGTCATGTGGAGATG-3′; Reverse Primer: 5′-CTGTTGCAGCCTTACTATACGCC-3′, β-Actin: forward: 5′-CTGGCACCCAGCACAATG-3′, and reverse 5′-GCCGATCCACACGGAGTACT-3′. The expression levels were standardized by one housekeeping gene, β-Actin in order to validate the result [48]. The comparative CT method was used to quantify mRNA expression and expressed as arbitrary units [49].

The protein lysates used for mass spectrometry analysis were resolved by SDS-PAGE (12% SDS-Tris-glycine gel). The gels were run under reducing conditions, and the resolved proteins were visualized with Coomassie stain. In addition, a portion of the protein lysates were resolved by SDS-PAGE and transferred to a polyvinylidene difluoride (PVDF) membrane for western blot analysis.

The proteins transferred were confirmed by staining the PVDF with 2 ml of amido black stain for 1 minute, and then washed 3 times with water. The membrane was washed thoroughly with TBS and was blocked with 5% milk in Tris Buffered Saline with Tween 20 (TBST) overnight with rocking at 4°C. The primary antibody TFRC, (Invitrogen 13–6800) was used at a dilution of 1:10000 for a time period of 1hour with rocking at room temperature. The secondary antibody, goat/anti-mouse IgG peroxidase conjugate was used appropriately at a dilution of 1:10000 in block solution and was incubated for 1 hour with rocking at room temperature. The ECL Perking Elmer detection kit system was used to visualize the reactive proteins on the blots. The quantification of TFRC protein levels was determined by normalization to glyceraldehyde-3-phosphate dehydrogenase (GAPDH).

### Functional annotation analytics

The functional annotations of the differentially abundant proteins were determined with bioinformatics tools. Subsequently, visual analytics tasks were conducted on the data sets obtained from the bioinformatics tools. A visual analytics tool allows the user to perform several human cognitive tasks on datasets through the integration of automated analysis, interaction and visualization [23,24,36,50]. The visual analytics approach will also enable user specific tasks or questions to be accomplished.

For the arsenic-regulated proteins, the Database for Annotation, Visualization and Integrated Discovery (DAVID) [27,28] was used to determine (i) the Universal Protein Resource (UniProt) tissue annotation [26]; and (ii) Gene Ontology annotation [31,51]. Data sets from (i) proteomic analysis; (ii) DAVID bioinformatics resources; and (ii) the Comparative Toxicogenomics Database were uploaded in Tableau Visual Analytics Software (Tableau Software Inc. Seattle, WA). The visualization and analysis designs were developed for several tasks that require blending of selected fields from multiple data sources. The International Protein Identifiers (IPI) served as the shared field.

Three tasks that were conducted using the visual analytics tools are reported in this article. The tasks were based on results from the DAVID Functional Annotation Tool. The results obtained from the Functional Annotation Tool were uploaded as a data source in Tableau Software. Subsequently, blending of the data sources was performed by selecting the appropriate fields including the shared field.

The first task was to identify arsenic regulated proteins that are annotated with UniProt tissue term “keratinocyte” or “Skin”. This task was accomplished by blending the tissue annotations from DAVID with the proteomics data set that we produced. In the second task, the proteomics data set was annotated and grouped by Gene Ontology (GO) terms for biological process. When the GO annotation is present, one or more GO terms could be mapped to the protein. For example: “GO:0006508~proteolysis,GO:0006511~ubiquitin-dependent protein catabolic process,GO:0009057~macromolecule catabolic process”. In our analytics, only the first GO identifier was selected to accomplish the annotation and grouping of the proteomics data set. To have access to the entire Gene Ontology terms for this research, the GO Terms and GO IDs were extracted from the full ontology file of the Gene Ontology Consortium (http://www.geneontology.org/GO.downloads.ontology.shtml). Providing the data set in a visual analytics tool will allow us also to identify GO terms that contain the word “arsenic”. In the third task, the tissue annotations for members of protein families in the proteomics data set were compared. The mapping of gene symbols to gene family descriptions was downloaded from the website of the Human Genome Organization Gene Nomenclature Committee (HGNC www.genenames.org). The gene family description data was uploaded to the visual analytics tool to group proteins by the tissue annotation profiles.

## Results

### Cytotoxicity assay (MTT Assay)

**Figure 1:**
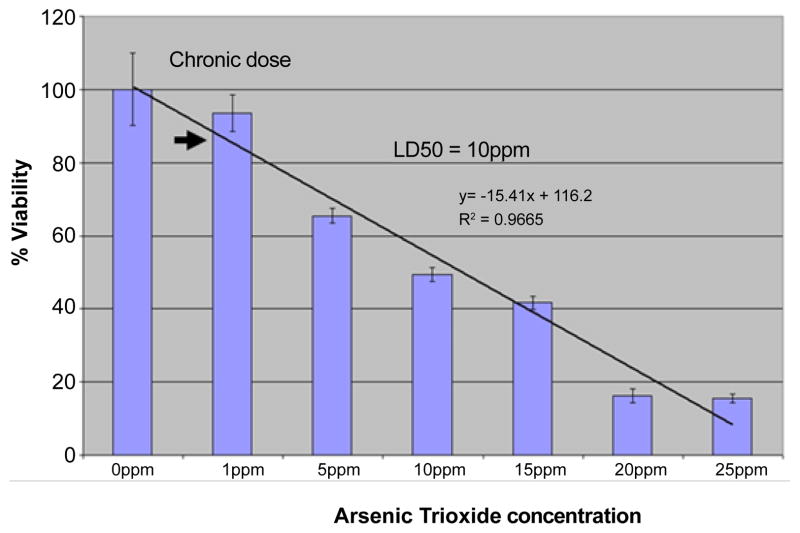
Cytotoxicity assay (MTT Assay) result To determine the LD10/LD 50 and estimate the minimal cytotoxic concentration that would be used to establish a chronic cell exposure condition. The exposure of HaCaT cells to arsenic had LD10 and LD50 values of 1ppm and 10ppm respectively. The LD10 dose result obtained from the initial cytoxicity assay was arbitrarily divided by 1/2 to get 0.5ppm of Arsenic that was used for chronic dosage treatment.

MTT assay shows that acute exposure of HaCaT cells to arsenic had LD10 and LD50 values of 1mg/L and 10mg/L respectively. The LD10 dose result obtained from the cytotoxicity assay (Figure 1) was arbitrarily divided by 1/2 to get 0.5 mg/L (0.5 ppm) of Arsenic that was used for chronic dosage treatment.

### Identification of proteins with differential abundance in HaCaT keratinocytes exposed to arsenic trioxide

**Figure 2:**
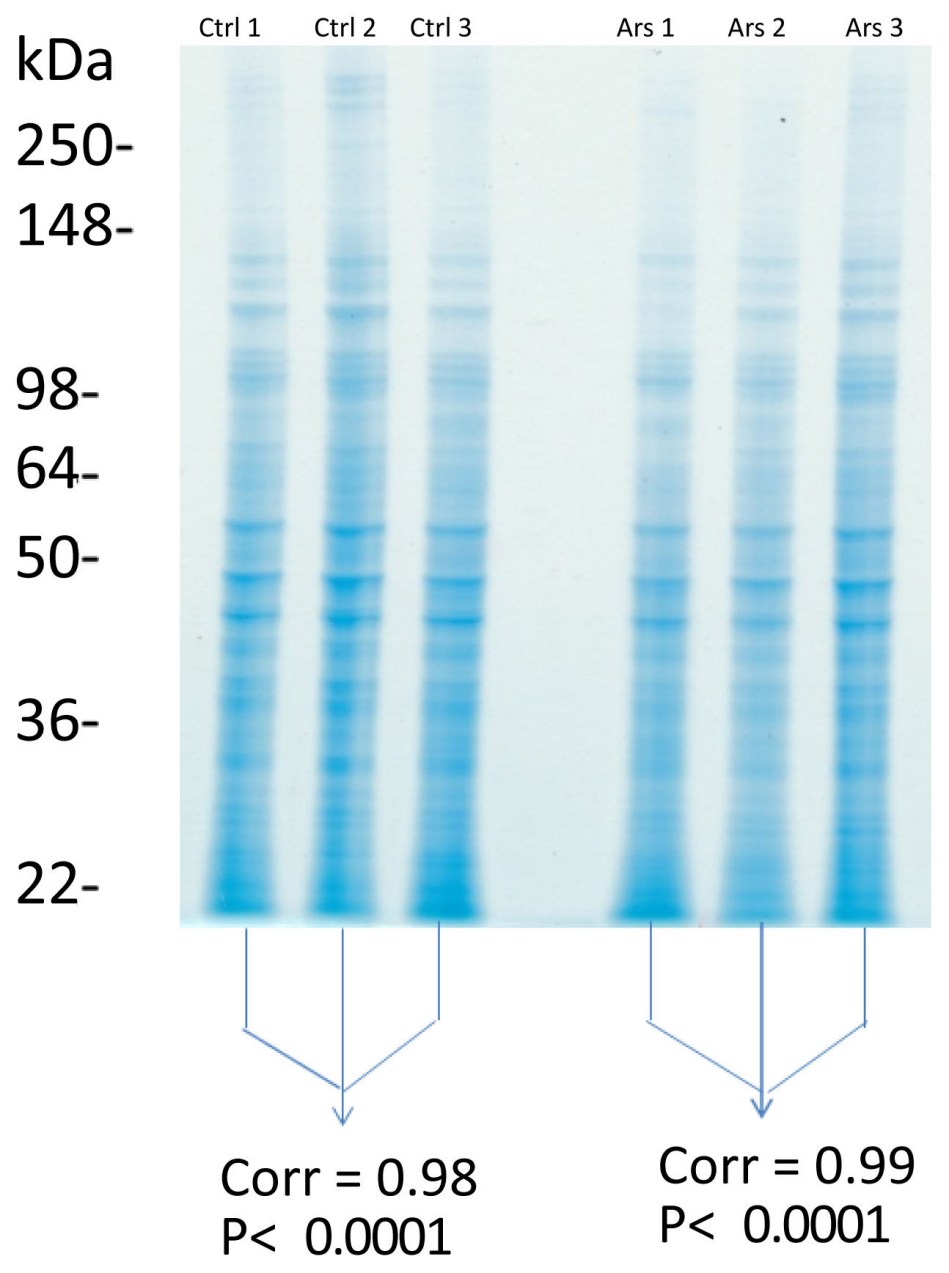
Demonstrating the reproducibility of the quantitative mass spectrometric method. Biological triplicate analyses of HaCaT cells not exposed to arsenic (passage control) and HaCaT cellexposed to 0.5 ppm arsenic for 8 passages, (passage in this study means sub-culturing after confluence). Ctrl=Control (no arsenic exposure, passage control) while Ars=Arsenic exposure (0.5ppm of arsenic for up-to 8 passages). Proteins were extracted from both exposed and unexposed HaCaT cell samples. An aliquot of protein lysate from each of the 6 culture plates was collected and resolved by SDS-PAGE/Coomassie. Each of the 6 gel lanes was entirely sliced into 24 bands, subjected to trypsin digestion, and peptides were analyzed by LC-MS/MS with a Thermo LTQ-XL mass spectrometer equipped with an Eksigent nano2D-LC. Proteins were identified with Mascot (95% confidence threshold). Pearson correlation analyses were performed to test reproducibility.

A total of 2164 proteins were identified from the arsenic exposed cells (1.0% false discovery rate using a decoy database from 192940 spectra). The control group yielded 2268 (0.8% false discovery rate using a decoy database from 198762 spectra). The Mascot results were uploaded into Scaffold 3 (version 3.00.01) for viewing the proteins and peptide information. A total of 1633 proteins were further analyzed after normalization (NSAF). A total of 173 unique proteins had significant increase or decrease in protein abundance when the HaCaT keratinocytes exposed to arsenic were compared to the HaCaT keratinocytes not exposed to arsenic (control). Transferrin receptor protein 1 (TFRC) had increased abundance in the cells. The reproducibility of the quantitative mass spectrometric method is demonstrated in Figure 2.

**Figure 3:**
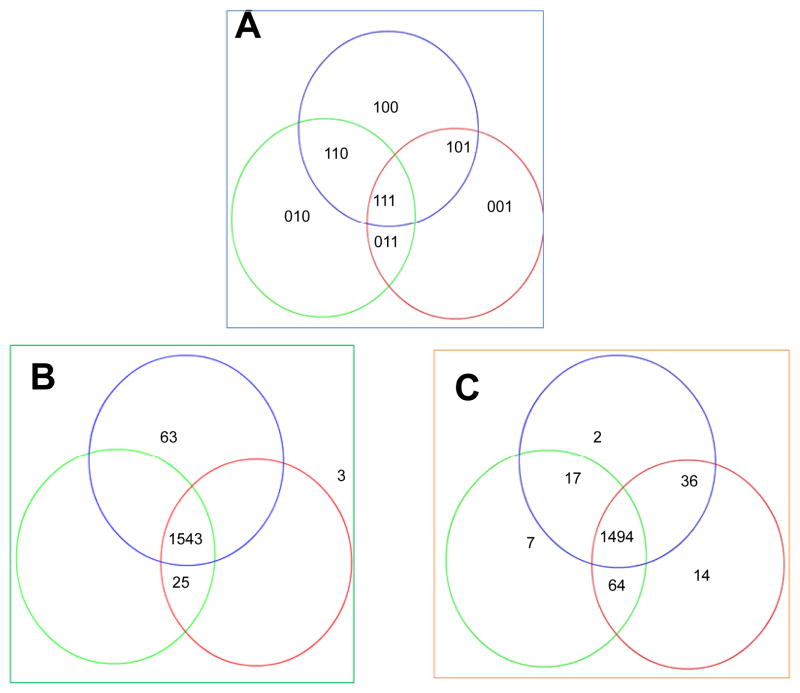
The reproducibility of mass spectrophotometer analysis for cells exposed to arsenic and those not exposed to arsenic A 3-digit binary code (A) was used to encode the availability for the three unexposed experiment set (B) and the three exposed experiment set (C). The data obtained from the two sets were then used to determine the agreement of availability of raw spectra data for all the six replicates. Of the total 1634 protein spots analyzed, 94.4% unexposed set and 91.4% in exposed set had presence of spectra in all replicates. In the case of the 173 differentially abundant proteins, there was presence of spectra in all the unexposed experiments while in two protein spots no spectra were detected. The probability that this distribution of presence/absence for the differentially abundant proteins was due to chance was tested using the Sign test. The probability was less than 0.0001.

The availability (presence or absence) of raw spectra data was determined for each biological replicate experiment. Since three biological replicates were done, a 3-digit binary code was used to encode the availability for the three exposed set and the three unexposed set. The data obtained from the two sets was then used to determine the agreement of availability of raw spectra data for all the six replicates. Of the total 1634 protein spots analyzed, 91.4% in exposed set and 94.4% unexposed set had presence of spectra in all replicates (Table 1). Within the 173 differentially abundant proteins only two proteins did not agree. The reproducibility of MS analysis is illustrated in Figure 3. The normalized MS result data and calculation formula are shown in the supplementary file.

**Figure 4:**
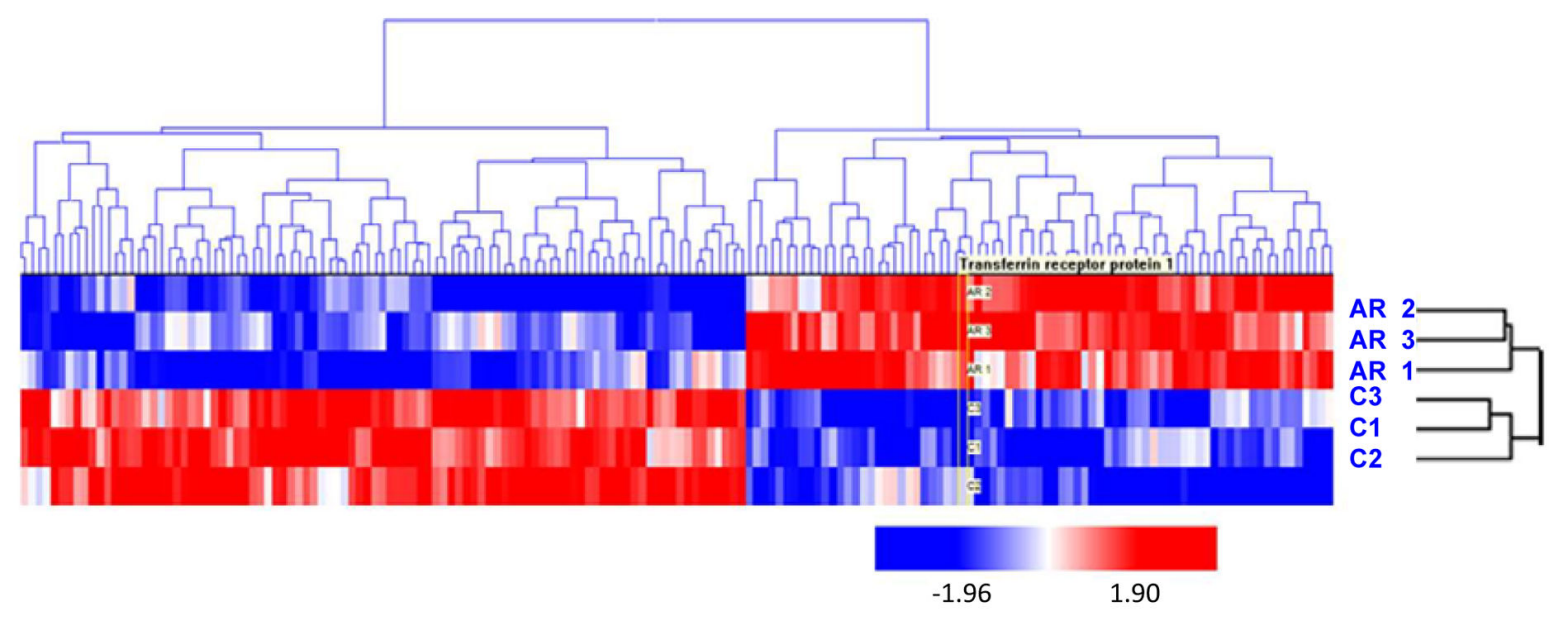
Hierarchical clustering of differentially abundant proteins in HaCaT keratinocytes exposed to arsenic. Clustered heat map of the 173 proteins with significant changes in abundance between arsenic treated (AR1, AR2, AR3) and untreated HaCaT cells (C1,C2,C3) as determined by t-test (p<0.05). An unsupervised cluster of significant proteins indicates a difference between the arsenic treated cells and the control cells. Red data points indicate increase protein abundance, while blue indicates decreaseabundance.

**Figure 5:**
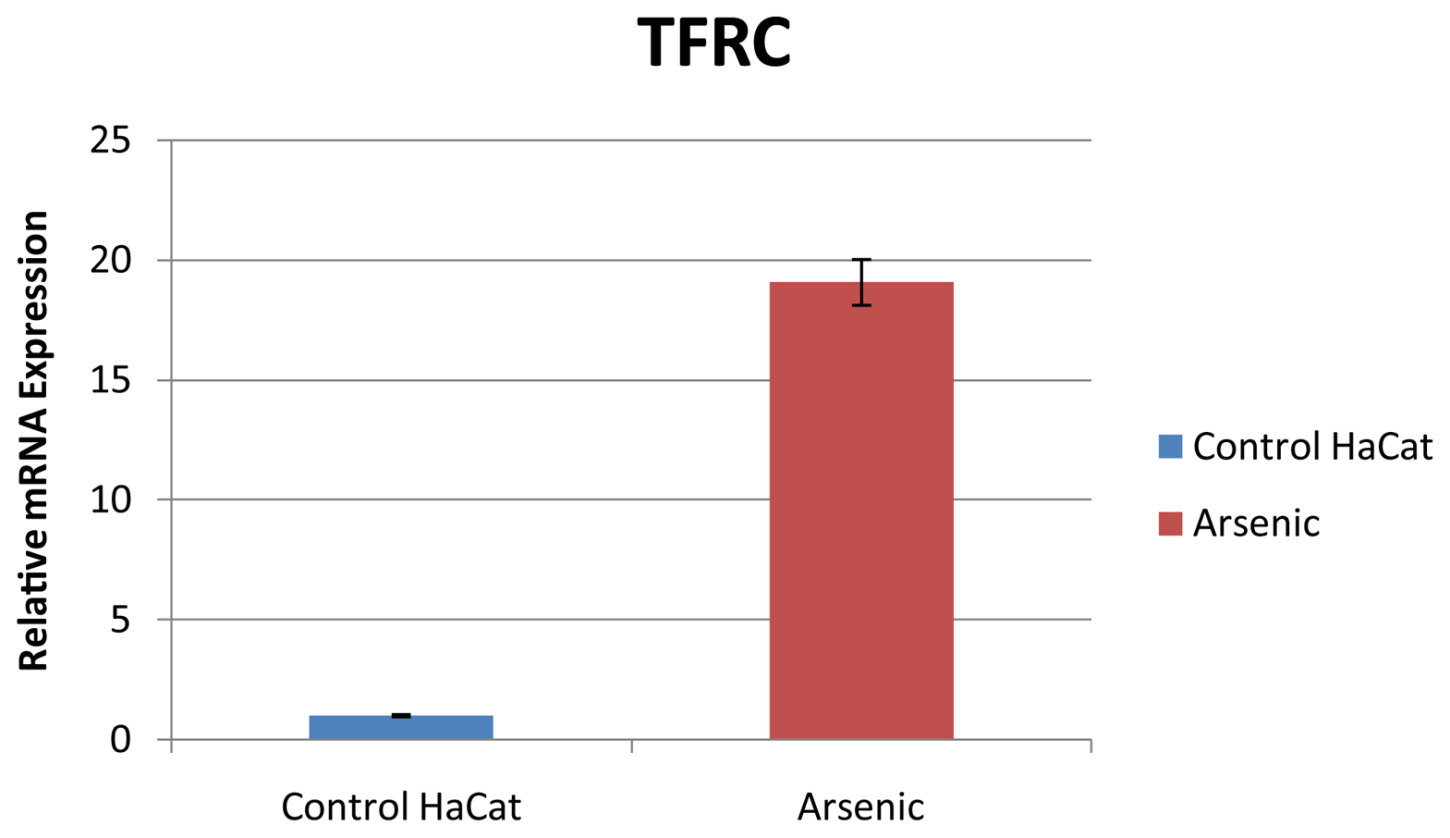
Relative mRNA expression induced by Arsenic in HaCaT cells. The relative quantification relates the PCR signal of TFRC transcript in arsenic exposed HaCaT cell compared to that of unexposed control HaCaT cells. Actin was used as the house keeping gene. TFRC was up-regulated by arsenic in the exposed HaCaT cells.

**Figure 6:**
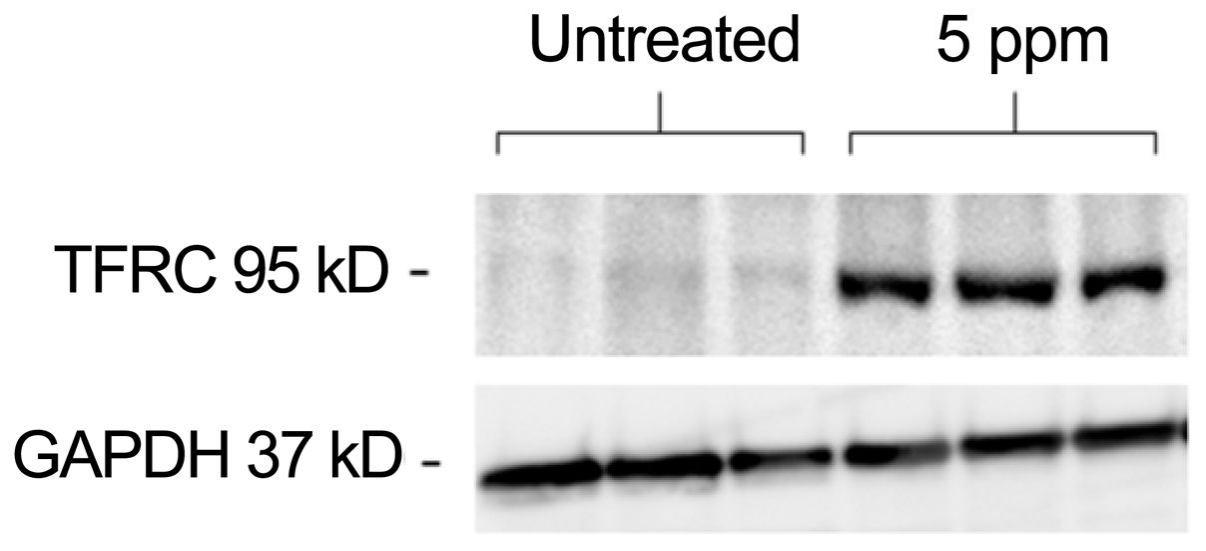
Western blotting analysis for TFRC proteins separated on a 12% reducing SDS gel. The proteins were transferred to a PVDF membrane and the size of the proteins seen 95 kDa and 37 kDa for GAPDH (House-keeping gene).

The 173 significant proteins, according to the lowest *p*-value from the t-test, were visually inspected by hierarchical clustering (Figure 4). Both RT-PCR and Western Blot experiments showed that arsenic induced a significant increase in abundance of TFRC in HaCaT cells treated with arsenic. In Figure 5, the relative quantification relates the PCR signal of TFRC transcript in arsenic exposed HaCaT cell compared to that of unexposed control HaCaT cells. The western blot result is illustrated in Figure 6. Two distinct proteins bands are noticeable with sizes of 95 kDa and 37 kDa which correspond with the TFRC and GAPDH (House-keeping gene) respectively.

In the treated HaCaT keratinocytes, 96 proteins showed increase in abundance while 77 proteins showed decrease in abundance. The complete list of proteins is found in the supplementary file. Additionally, a visual analytics resource for exploring and user-defined analysis is available at http://public.tableausoftware.com/profile/#!/vizhome/arsenic_hacat/abstract. We recognize that users of our dataset could prefer to set other cutoff ratio when comparing the relative abundance of proteins between untreated and treated cells. Therefore, we have included filter with sliding bar for values of both the t-test and the cut-off ratio. The interaction with the dataset is available at http://public.tableausoftware.com/profile/#!/vizhome/arsenic_hacat/all_reg_proteins

### Identification of arsenic regulated proteins annotated with terms: keratinocyte or skin

Protein annotation performed using DAVID with proteins annotated with skin tissue as the filter revealed two clusters of 20 up-regulated and 34 down-regulated proteins (Table 2). Two groups of regulated proteins were 5 up-regulated and 5 down-regulated proteins (Table 3). The up-regulated proteins include Calnexin precursor, Isoform 7 of Filamin-B, heterogeneous nuclear, ribonucleoprotein L isoform A, Keratin, type II cytoskeletal 7, Keratin, type I cytoskeletal 19. The down-regulated protein groups were Involucrin, Protein S100-A2, Protein S100-A8, Protein S100-A9, Isoform Long of 14-3-3 protein beta/alpha.

### Grouping of arsenic regulated proteins by gene ontology biological process terms

**Figure 7:**
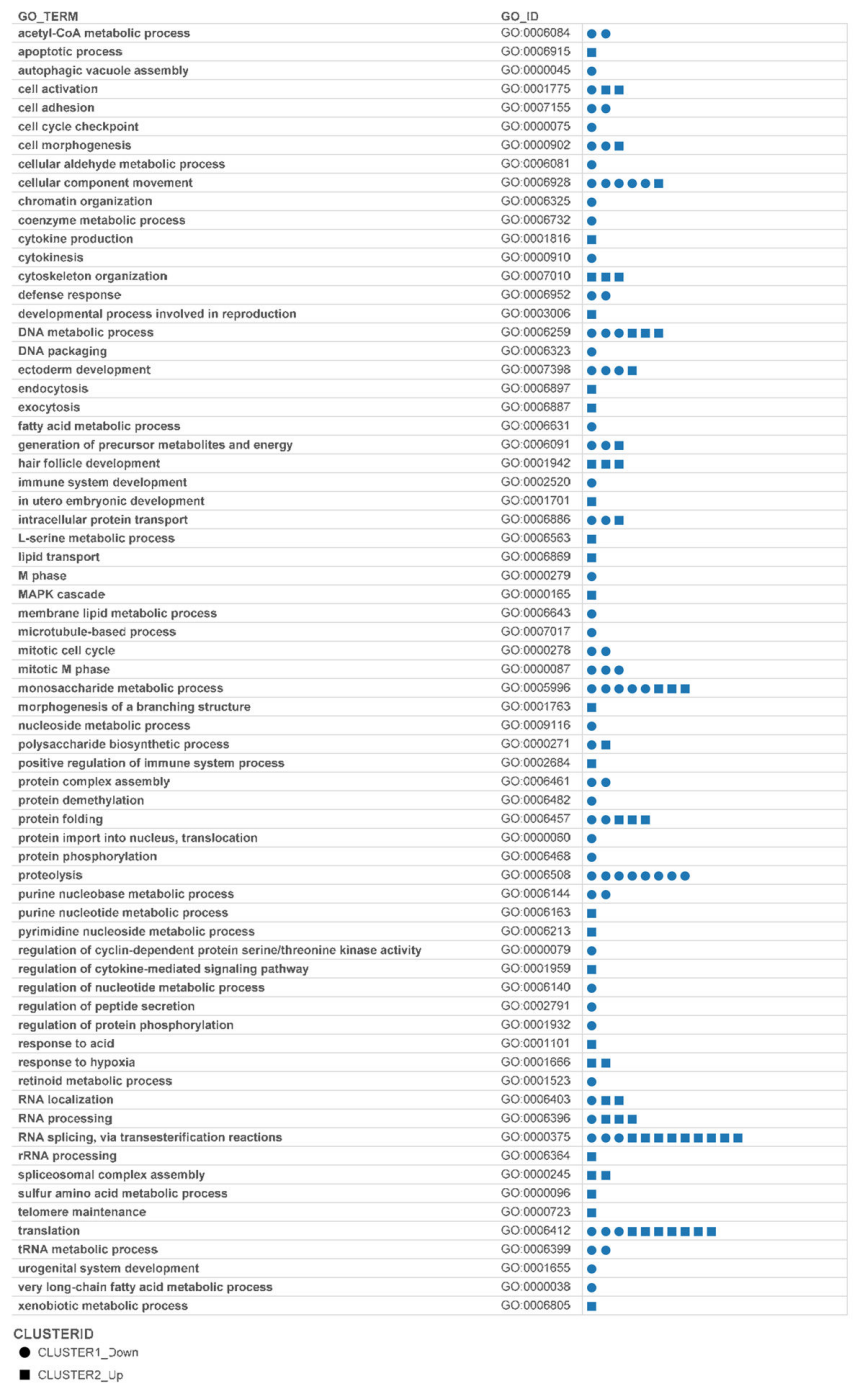
Grouping of arsenic regulated proteins by Gene Ontology biological process terms. We observed 69 Gene Ontology Biological Process terms which were associated with 148 arsenic regulated proteins. The visualization allows from comparison of the number of proteins with increased abundance versus proteins with decreased abundance. Visualization can be accessed at http://public.tableausoftware.com/profile/#!/vizhome/arsenic_hacat/GO_anno_viz

A total of 69 Gene Ontology Biological Process terms were associated with 148 arsenic regulated proteins (Figure 7). The visualization allows from comparison of the number of proteins with increase in abundance versus proteins with decrease in abundance. Numerous decisions can be made from the visualization. However, we decided to identify other arsenic regulated proteins that are grouped in the same biological process as TFRC (IPI00022462). ATPase, Na+/K+ transporting, beta 1 polypeptide (ATP1B1, IPI00747849) was the only other protein with “response to hypoxia” Gene Ontology biological process.

### Proposing major pathways for arsenic toxicity response

The Gene Ontology Biological Process terms were searched for terms that include the word “metabolic” so as to identify major pathways for arsenic toxicity. A total of 33 proteins (11 increased abundance and 22 decreased abundance) associated with 18 metabolic process terms were identified (Tables 4 and 5). Twenty-five of the protein names included the “ase” indicating enzyme function. There were four protein names that included “mitochondrial”. Since arsenic binds with sulfur, we mined for terms or protein names with sulfur to increase our chances of discovering arsenic-interacting proteins. We reason that the presence of these proteins in our dataset will help validate the relevance of the list of protein whose abundance is altered by arsenic. Two proteins were identified to have sulfur in their Gene Ontology biological process Term or Protein name. The Glutamate--cysteine ligase catalytic subunit (GCLC), the only protein annotated with the term sulfur amino acid metabolism process, had increased abundance in arsenic-exposed HaCaT keratinocytes. Succinate dehydrogenase [ubiquinone] iron-sulfur subunit, mitochondrial precursor (SDHB), had decreased abundance in arsenic-exposed HaCaT keratinocytes. The proteins mapped to metabolic process terms as well as to arsenic-regulation information are candidates for evaluating perturbation of metabolic pathways by arsenicals. Our discussion will focus on GCLC and SDHB as examples of major pathways involved in arsenic toxicity response.

### Comparison of tissue annotations for members of protein families

**Figure 8:**
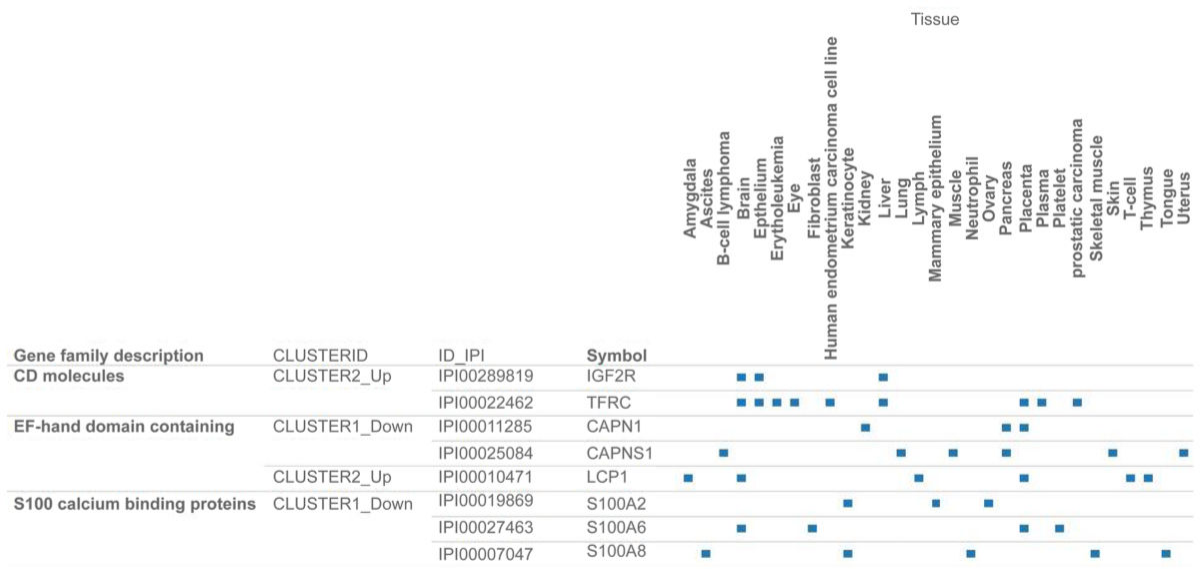
An integration of datasets on proteomic response, tissue annotation, and gene family description for three gene families. TFRC which forms part of up-regulated Cluster of Differentiation (CD) molecules was annotated with terms: brain, erythroleukemia, eye, human endometrium carcinoma cell line, kidney, pancreas, placenta and prostatic carcinoma. The down-regulated S100 calcium binding proteins were S100A2, S100A6, S1008 with A100A2 and S100A8 annotated with term keratinocyte. Visualization can be accessed at http://public.tableausoftware.com/profile/#!/vizhome/arsenic_hacat/family2tissue

A total of 165 arsenic regulated proteins were annotated with at least one tissue term with a total of 144 terms associated with the proteins. To continue our knowledge-building on TFRC, we decided to include the gene family that includes the TFRC. Figure 8 presents a comparison of tissue annotation profiles and integration of datasets on proteomic response, tissue annotation, and gene family description for three protein families. The protein families are Cluster of Differentiation (CD) molecules; EF-hand domain containing and S-100 calcium binding protein. The CD molecules family included TFRC and Insulin-Growth Factor Receptor 2 (IGF2R). TFRC is annotated with the following 8 terms: brain, erythroleukemia, eye, human endometrium carcinoma cell line, kidney, pancreas, placenta, and prostatic carcinoma. Though three S100 calcium binding proteins (S100A2, S100A6 and S100A8) were down-regulated, only S100A2 and S100A8 were annotated with keratinocyte term.

## Discussion

Arsenic is extensively absorbed in the skin keratinocytes where the toxicant induces changes to the morphology and physiology of the cell [52,53]. We have used quantitative label-free mass spectrometry to determine the arsenic trioxide induced changes in abundance of proteins in HaCaT keratinocyte model. Comparing the availability of spectra data for protein spots reveal over 90% agreement in the availability (presence or absence) of raw spectra data for the exposed set and the unexposed set. This high value of agreement combined with identification of known arsenic perturbed proteins indicates that the data obtained is reliable.

A total of 173 proteins were differentially abundant after arsenic insult when comparing arsenic exposed versus unexposed HaCaT keratinocytes. The list of all the significant proteins with associated proteomic data are provided as supplementary file and online resources. A visual analytical integration strategy of functional annotations including UniProt Tissue and Gene Ontology [30,54] was developed for several reasons. The integration helped to identify 10 arsenic regulated proteins already known to function in keratinocytes (Table 2). These categorized lists of arsenic-regulated proteins could be investigated for roles in arsenic induced diseases. These proteins are also candidates for inclusion in curated toxicogenomics databases such as the Comparative Toxicogenomics Database [55].

Gene Ontology (GO) biological process classification of differentially abundant proteins classified 149 proteins into 69 biological process terms. The distribution of arsenic regulated (up or down) is provided to enable follow-up research on biological processes (Figure 7). We have also provided supplementary resources to aid the several cognitive tasks of our datasets; including a visual analytics resource for exploring the Gene Ontology terms associated with 40,909 Gene Ontology Identifiers. We identified a need to expand the number of genes annotated with the GO term response to arsenic-containing substance (GO:0046685).

One of our interests was to identify proteins annotated with tissue term keratinocyte. Therefore, this discussion includes the relevance of two groups of regulated proteins consisting of 5 up-regulated and 5 down-regulated proteins (Table 3). The up-regulated proteins include Calnexin precursor; Isoform 7 of Filamin-B; heterogeneous nuclear; ribonucleoprotein L isoform A; Keratin, type II cytoskeletal 7; and Keratin, type I cytoskeletal 19. The down-regulated proteins were Involucrin, Protein S100-A2, Protein S100-A8, Protein S100-A9, and Isoform Long of 14-3-3 protein beta/alpha.

Calnexin is an endoplasmic reticulum (ER) transmembrane chaperone involved in translocation, in protein folding, and in the quality control of newly synthesized polypeptides [56–58]. There are no reported studies on direct calnexin-arsenic interaction and since calnexin plays critical cellular roles, any perturbation of its expression may have detrimental consequences [59,60]. Filamins (FLNs) are large actin-binding, cross-linking and scaffolding proteins. They stabilize the membrane by anchoring the transmembrane proteins and are implicated in signal transduction, through interaction with ion channels, receptors and signaling proteins [61,62]. Mutations and perturbation in the FLNA and FLNB genes are known to cause different developmental disorders in humans, such as bone anomalies, periventricular heterotopia, aortic dissection and aneurysm [62,63]. Tumor marker proteins including stress-activated protein kinase/c-Jun N-terminal kinase (SAPK/JNK) that belongs to the mitogen activated protein kinase (MAPK) are known to be up-regulated in HaCaT cells exposed to arsenic [63]. SAPK/JNK and MAPK are associated with reactive oxygen species induction and other signal transduction. Filamin links two MAPKKs together and promote JNK activation [64].

Heterogeneous nuclear ribonucleoprotein L isoform A (HNRNPL) is a splicing regulatory protein [65] which is up-regulated in our work. It has also been implicated in other cancers such as esophageal squamous cell carcinoma [66]. Understanding the molecular mechanisms of HNRNPL induction and its role during keratinocyte transformation by arsenic will be important since they function as a docking protein for DNA, RNA, and transcriptional molecules [67].

Keratin, type II cytoskeletal 7 and Keratin, type I cytoskeletal 19 were up-regulated in this study. Keratins are major components of structural proteins of the epidermis and are one of the most abundant proteins in epithelial cells. Alterations in keratin expression are associated with the development of skin pathology, including hyperkeratosis, and skin cancers [68,69]. Keratinocytes in the epidermis express a differentiation-specific set of type I and type III keratins which form a stable network and contribute to keratinocyte mechanical properties [70]. Mapping of the cytokeratin profile of the special type of skin pathology may be crucial in typing of the stages of the disease as has been attempted with breast carcinoma [71].

Involucrin (IVL) is a marker of keratinocyte differentiation and its down regulation as observed in our study has been related to various disease conditions. Involucrin function was perturbed in skin squamous cell carcinoma (SCC) [72], and cervical cancer [73]. The protein levels were down-regulated by human papilloma virus (HPV) oncogenes in human foreskin keratinocyte (HFK) cells [74]. Arsenate suppresses IVL expression, and, keratinocyte programming, probably by altering the transcription factors AP1 (activating protein-1) and AP2 (activating protein-2) [75].

S100 proteins are a group of proteins that are involved in the regulation of a number of cellular processes such as cell cycle progression and differentiation. They are found in the cytoplasm and sometimes in the nucleus of a wide range of cells. The tissue annotation profile in Figure 6 revealed that the down-regulated S100 calcium binding proteins, especially A100A2 and S100A8 were associated with keratinocytes. We observed down-regulation of Protein S100-A2, Protein S100-A8, and Protein S100-A9. Protein S100-A8 and Protein S100-A9 are suggested as markers for early diagnosis of necrotizing enterocolitis in neonates [76]. Protein S100-A2, is an EF hand calcium-binding protein, that is perturbed in several cancers and is also a TGF-β (transforming growth factor-β)-regulated gene in melanoma and lung cancer cells [77]. S100A2 was identified as putative tumor suppressor gene in human breast cancer cell lines [78]. In other words its suppression by arsenic will allow tumor cell to replicate unabated. Altered expression and chromosomal rearrangements of S100 proteins have been implicated in breast cancer [79].

Isoform Long of 14-3-3 protein beta/alpha, though down-regulated in this study was previously observed to be over expressed in cyst fluid from cyst-associated renal cell carcinoma. Its differential expression makes it a potential urinary biomarker for renal cell carcinoma [80]. The perturbation of Isoform Long of 14-3-3 protein beta/alpha may suggest its involvement in HaCaT cells carcinogenesis or response to arsenic and it will be very interesting to understand its roles more.

We previously predicted that arsenic will interact with TFRC based on the presence of vicinal cysteines [80]. Thus, after the observed up-regulation of Transferrin receptor protein 1 (TFRC) in the proteomics data, we conducted western blot assay (Figure 4) to build additional evidence that arsenic exposure increases the abundance of TFRC and possibly increase iron transport into keratinocytes. A comparison of tissue annotation profiles and integration of datasets on proteomic response, tissue annotation (Figure 6) showed that TFRC, an up-regulated Cluster of Differentiation (CD) molecule, is associated with the brain, erythroleukemia, eye, human endometrium carcinoma cell line, kidney, pancreas, placenta and prostatic carcinoma. A previous study had reported increased expression of transferrin receptor in cancer cells than in normal cells [81]. TFRC has been suggested as a potential biomarker in the diagnosis of early onset of colorectal cancer [82]. A proteomics analysis showed that TFRC is highly expressed in inflammatory breast cancer [83].

We observed in our previous study that low dose arsenic triggers proliferation of HaCaT cells [84]. The cell proliferation could be linked to the expression of TFRC. This observation was corroborated by Ryschich et al. who reported TFRC as a membrane-bound protein expressed in larger amounts in proliferating, human pancreatic cancer cells than in quiescent cells [85]. We have demonstrated increased expression of TFRC in HaCaT cells exposed to arsenic trioxide using proteomic, transcriptomics and western blot. Arsenic induces skin color disorders such as hyper and hypopigmentation [86]. TFRC is a carrier protein for iron which is a crucial component of melanin. TFRC may also be involved in the transfer of melanin from melanocytes to other keratinocytes using the transferrin receptor-mediated endocytosis pathway [87,88]. Increased melanocyte dendricity is correlated with hyperpigmentation [38]. Disruption of the iron transfer system by arsenic could lead to skin discoloration pathologies such as hyperpigmentation and hypopigmentation.

The transferrin receptor expression is modulated by hypoxia. In rats, hypoxia increases expression of transferrin receptor leading to increased cellular uptake of iron in the pineal gland [89]. Also in humans, hypoxia is a side effect of arsenic trioxide treatment [90]. The skin is normally mildly hypoxic ranging from 1.5% to 5% oxygen [90]. The modulation of hypoxic levels in the keratinocytes by arsenic leading to accumulation of iron species warrants further research.

A total of 33 proteins (11 increased abundance and 22 decreased abundance) associated with 18 metabolic process terms were identified (Table 4). The inclusion of proteins involved in the mitochondrial metabolic function is consistent with arsenic suppressing mitochondrion function through binding with thiol-containing enzymes on the mitochondrion membrane [91]. The Glutamate--cysteine ligase catalytic subunit (GCLC) is one of the oxidative stress defense genes regulated by Transcription factor NF-E2-related factor 2 (Nrf2) [92]. In HaCaT keratinocytes, *Gclc* gene expression is induced by arsenite [93]. This prior finding provided independent confirmation of the differentially abundant gene list reported here. Succinate dehydrogenase [ubiquinone] iron-sulfur subunit, mitochondrial precursor (SDHB) had decreased abundance after arsenic exposure compared to unexposed cells (Table 4). The SDHB is part of the catalytic domain of succinate dehydrogenase, an enzyme complex that functions in the citric acid cycle and the electron transport chain [94]. We inferred from the presence of sulfur in the subunit that arsenic could bind to SDHB and interfere with its function. In fact, defect in SDHB induces tumor formation due to succinate accumulation [95,96].

In conclusion, a list of 173 protein altered by arsenic trioxide were grouped using three major functional annotations covering tissue localization, biological process and protein family. A possible explanation for hyperpigmentation pathologies observed in arsenic toxicity is that arsenic exposure leads to increased iron uptake in the normally hypoxic human skin. The proteins mapped to metabolic process terms and differentially abundant are candidates for evaluating metabolic pathways perturbed by arsenicals.

## Supplementary Material

Supplementary File

## Figures and Tables

**Table 1:** Agreement on availability of raw spectra data in biological replicates.

Binary Code for Unexposed	Binary Code for Exposed						
	001	010	011	100	101	110	111
000							3
011	3		4		6	3	9
100	1	1	13		3	2	43
111	10	6	47	2	27	12	1439

A 3-digit binary code was used to encode the availability for the three exposed replicates set and the three unexposed replicates set using the raw spectra data. Presence and absence of spectra are encoded as 1 and 0 respectively.

**Table 2:** Arsenic regulated proteins annotated with the skin tissue.

	Up-Regulated		Down Regulated
ID_IPI	Protein Name	ID_IPI	Protein Name
IPI00003377	Isoform 1 of Splicing factor, arginine/serine-rich 7	IPI00001580	Isoform 1 of FYVE and coiled-coil domain-containing protein 1
IPI00009407	Dolichyl-diphosphooligosaccharide--protein glycosyltransferase subunit DAD1	IPI00003783	Dual specificity mitogen-activated protein kinase kinase 2
IPI00010271	Isoform A of Ras-related C3 botulinum toxin substrate 1 precursor	IPI00005162	Actin-related protein 2/3 complex subunit 3
IPI00011118	Ribonucleoside-diphosphate reductase M2 subunit	IPI00007694	Isoform 1 of Protein phosphatase methylesterase 1
IPI00012750	40S ribosomal protein S25	IPI00009841	CDNA FLJ31747 fis, clone NT2RI2007377, highly similar to RNA-BINDING PROTEIN EWS
IPI00015954	GTP-binding protein SAR1a	IPI00011107	Isocitrate dehydrogenase [NADP], mitochondrial precursor
IPI00021840	40S ribosomal protein S6	IPI00011578	Isoform 1 of Neuroplastin precursor
IPI00023048	Elongation factor 1-delta	IPI00011692	Involucrin
IPI00023101	Protein RCD1 homolog	IPI00013933	Isoform DPI of Desmoplakin
IPI00056334	protein kinase C, delta binding protein	IPI00025084	Calpain small subunit 1
IPI00215768	Glutamate--cysteine ligase catalytic subunit	IPI00216318	Isoform Long of 14-3-3 protein beta/alpha
IPI00219365	Moesin	IPI00219301	Myristoylated alanine-rich C-kinase substrate
IPI00221088	40S ribosomal protein S9	IPI00219526	Isoform 1 of Phosphoglucomutase-1
IPI00290279	Isoform Long of Adenosine kinase	IPI00240675	programmed cell death 4 isoform 2
IPI00293260	Isoform 1 of DnaJ homolog subfamily C member 10 precursor	IPI00293464	DNA damage-binding protein 1
IPI00295992	Isoform 2 of ATPase family AAA domain-containing protein 3A	IPI00307200	Switch-associated protein 70
IPI00329629	DnaJ homolog subfamily C member 7	IPI00412880	Isoform 1 of Histone-arginine methyltransferase CARM1
IPI00384444	Keratin, type I cytoskeletal 14	IPI00001580	Isoform 1 of FYVE and coiled-coil domain-containing protein 1
IPI00414676	Heat shock protein HSP 90-beta	IPI00003783	Dual specificity mitogen-activated protein kinase kinase 2
IPI00449049	Poly [ADP-ribose] polymerase 1	IPI00005162	Actin-related protein 2/3 complex subunit 3
		IPI00007694	Isoform 1 of Protein phosphatase methylesterase 1
		IPI00009841	CDNA FLJ31747 fis, clone NT2RI2007377, highly similar to RNA-BINDING PROTEIN EWS
		IPI00011107	Isocitrate dehydrogenase [NADP], mitochondrial precursor
		IPI00011578	Isoform 1 of Neuroplastin precursor
		IPI00011692	Involucrin
		IPI00013933	Isoform DPI of Desmoplakin
		IPI00025084	Calpain small subunit 1
		IPI00216318	Isoform Long of 14-3-3 protein beta/alpha
		IPI00219301	Myristoylated alanine-rich C-kinase substrate
		IPI00219526	Isoform 1 of Phosphoglucomutase-1
		IPI00240675	programmed cell death 4 isoform 2
		IPI00293464	DNA damage-binding protein 1
		IPI00307200	Switch-associated protein 70
		IPI00412880	Isoform 1 of Histone-arginine methyltransferase CARM1

Protein annotation was performed using DAVID with proteins annotated with skin tissue as the filter, and two clusters of 20 up-regulated and 34 down-regulated proteins were obtained

**Table 3:** Arsenic regulated proteins annotated with keratinocyte term.

CLUSTERID	Symbol	ID_IPI	Protein Name	Tissue Annotation
CLUSTER1_Down	IVL	IPI00011692	Involucrin	Keratinocyte, Skin
	S100A2	IPI00019869	Protein S100-A2	Keratinocyte, Mammary epithelium, Ovary
	S100A8	IPI00007047	Protein S100-A8	Ascites, Keratinocyte, Neutrophil, Skeletal muscle, Tongue
	S100A9	IPI00027462	Protein S100-A9	Keratinocyte, Liver, Lung
	YWHAB	IPI00216318	Isoform Long of 14-3-3 protein beta/alpha	Brain, Colon carcinoma, Keratinocyte, Platelet, Skin
CLUSTER2_Up	CANX	IPI00020984	Calnexin precursor	B-cell lymphoma, Epithelium, Fibroblast, Keratinocyte, Kidney, Liver, Lymph, Placenta, Platelet
	FLNB	IPI00477536	Isoform 7 of Filamin-B	Aortic endothelium, Endometrial tumor, Endometrium carcinoma cell line, Endothelial cell, Epithelium, Fetal brain, Keratinocyte, Placenta, Skeletal muscle, Thyroid,
	HNRNPL	IPI00027834	heterogeneous nuclear ribonucleoprotein L isoform a	Brain, Cajal-Retzius cell, Fetal brain cortex, Keratinocyte, Pancreatic cancer, Synovial membrane tissue, Uterus
	KRT7	IPI00306959	Keratin, type II cytoskeletal 7	Colon carcinoma, Epithelium, Keratinocyte, Mammary cancer, Mesothelium, Ovarian carcinoma, Pancreas, PCR rescued clones, Placenta,
	KRT19	IPI00479145	Keratin, type I cytoskeletal 19	Keratinocyte, Lymph node, Mammary gland, Pancreas, Peripheral blood leukocyte, Placenta

Using DAVID, categorization for annotation with keratinocytes yielded two clusters of 5 up-regulated and 5 down-regulated proteins.

**Table 4:** Proteins in metabolic processes with increased abundance to arsenic trioxide.

CLUSTERID	GO_TERM	GO_ID	ID_IPI	Protein Name
CLUSTER2_Up	DNA metabolic process	GO:0006259	IPI00011118	Ribonucleoside-diphosphate reductase M2 subunit
CLUSTER2_Up	DNA metabolic process	GO:0006259	IPI00306959	Keratin, type II cytoskeletal 7
CLUSTER2_Up	DNA metabolic process	GO:0006259	IPI00026087	Barrier-to-autointegration factor
CLUSTER2_Up	L-serine metabolic process	GO:0006563	IPI00019178	Phosphoserine phosphatase
CLUSTER2_Up	monosaccharide metabolic process	GO:0005996	IPI00219616	Ribose-phosphate pyrophosphokinase 1
CLUSTER2_Up	monosaccharide metabolic process	GO:0005996	IPI00000684	Isoform AGX2 of UDP-N-acetylhexosamine pyrophosphorylase
CLUSTER2_Up	monosaccharide metabolic process	GO:0005996	IPI00009790	6-phosphofructokinase type C
CLUSTER2_Up	purine nucleotide metabolic process	GO:0006163	IPI00290279	Isoform Long of Adenosine kinase
CLUSTER2_Up	pyrimidine nucleoside metabolic process	GO:0006213	IPI00029631	Enhancer of rudimentary homolog
CLUSTER2_Up	sulfur amino acid metabolic process	GO:0000096	IPI00215768	Glutamate--cysteine ligase catalytic subunit
CLUSTER2_Up	xenobiotic metabolic process	GO:0006805	IPI00012069	NAD

**Table 5:** Proteins in metabolic processes with decreased abundance to arsenic trioxide

CLUSTERID	GO_TERM	GO_ID	ID_IPI	Protein Name
CLUSTER1_Down	acetyl-CoA metabolic process	GO:0006084	IPI00096066	Succinyl-CoA ligase [GDP-forming] beta-chain, mitochondrial precursor
CLUSTER1_Down	acetyl-CoA metabolic process	GO:0006084	IPI00294911	Succinate dehydrogenase [ubiquinone] iron-sulfur subunit, mitochondrial precursor
CLUSTER1_Down	cellular aldehyde metabolic process	GO:0006081	IPI00011107	Isocitrate dehydrogenase [NADP], mitochondrial precursor
CLUSTER1_Down	coenzyme metabolic process	GO:0006732	IPI00184821	Bifunctional coenzyme A synthase
CLUSTER1_Down	DNA metabolic process	GO:0006259	IPI00013871	Ribonucleoside-diphosphate reductase large subunit
CLUSTER1_Down	DNA metabolic process	GO:0006259	IPI00018349	DNA replication licensing factor MCM4
CLUSTER1_Down	DNA metabolic process	GO:0006259	IPI00184330	DNA replication licensing factor MCM2
CLUSTER1_Down	fatty acid metabolic process	GO:0006631	IPI00024993	Enoyl-CoA hydratase, mitochondrial precursor
CLUSTER1_Down	membrane lipid metabolic process	GO:0006643	IPI00005745	Serine palmitoyltransferase 1
CLUSTER1_Down	monosaccharide metabolic process	GO:0005996	IPI00550364	Phosphoglucomutase-2
CLUSTER1_Down	monosaccharide metabolic process	GO:0005996	IPI00479186	Isoform M2 of Pyruvate kinase isozymes M1/M2
CLUSTER1_Down	monosaccharide metabolic process	GO:0005996	IPI00219526	Isoform 1 of Phosphoglucomutase-1
CLUSTER1_Down	monosaccharide metabolic process	GO:0005996	IPI00073772	Fructose-1,6-bisphosphatase 1
CLUSTER1_Down	monosaccharide metabolic process	GO:0005996	IPI00063408	dehydrogenase E1 and transketolase domain containing protein 1
CLUSTER1_Down	nucleoside metabolic process	GO:0009116	IPI00219617	Isoform 1 of Ribose-phosphate pyrophosphokinase 2
CLUSTER1_Down	purine nucleobase metabolic process	GO:0006144	IPI00025273	Isoform Long of Trifunctional purine biosynthetic protein adenosine-3
CLUSTER1_Down	purine nucleobase metabolic process	GO:0006144	IPI00029079	GMP synthase
CLUSTER1_Down	regulation of nucleotide metabolic process	GO:0006140	IPI00220578	Guanine nucleotide-binding protein G
CLUSTER1_Down	retinoid metabolic process	GO:0001523	IPI00026663	Aldehyde dehydrogenase 1A3
CLUSTER1_Down	tRNA metabolic process	GO:0006399	IPI00004860	Isoform Complexed of Arginyl-tRNA synthetase, cytoplasmic
CLUSTER1_Down	tRNA metabolic process	GO:0006399	IPI00216951	Aspartyl-tRNA synthetase, cytoplasmic
CLUSTER1_Down	very long-chain fatty acid metabolic process	GO:0000038	IPI00019912	Peroxisomal multifunctional enzyme type 2

A search for candidate proteins involved in metabolic pathways of arsenic toxicity response yielded a total of 33 proteins (11 increased abundance and 22 decreased abundance) associated with 18 metabolic process terms were identified
